# Copolymerization of Ethylene with Selected Vinyl Monomers Catalyzed by Group 4 Metal and Vanadium Complexes with Multidentate Ligands: A Short Review

**DOI:** 10.3390/polym13244456

**Published:** 2021-12-19

**Authors:** Marzena Białek, Julia Fryga

**Affiliations:** Institute of Chemistry, University of Opole, Oleska 48, 45-052 Opole, Poland; julia.herud@gmail.com

**Keywords:** post-metallocene catalyst, copolymerization, ethylene, 1-olefin, styrenic monomer, α,ω-alkenol

## Abstract

This paper gives a short overview of homogeneous post-metallocene catalysts based on group 4 metal and vanadium complexes bearing multidentate ligands. It summarizes the catalytic behavior of those catalysts in copolymerization of ethylene with 1-olefins, with styrenic monomers and with α,ω-alkenols. The review is focused on finding correlations between the structure of a complex, its catalyst activity and comonomer incorporation ability, as well as the microstructure of the copolymer chains.

## 1. Introduction

According to statistics, the global production of plastics in 2019 reached 368 million tons [1]. The production of polyethylene is estimated to account for about one third of all plastics manufactured, of which nearly 80% is formed in the catalytic processes [2]. The catalysts based on transition metal compounds were, for the first time, employed in the ethylene polymerization in the first half of 1950s. These were the Philips supported chromium catalyst and Ziegler–Natta catalysts comprising titanium chlorides and organoaluminium compounds which were converted into supported systems in time [3]. These multi-site catalysts produce polymers which are mixtures of macromolecules with different lengths and, in the case of copolymers, also with different compositions. Then, single-site metallocene and constrained geometry catalysts (CGC) were developed, which made it possible to synthesize polyolefins with more uniform macromolecules with narrow molecular weight distribution and more homogeneous comonomer distribution [4,5,6]. Moreover, they afford the incorporation of bulky olefins into polyethylene chains, as well as allowing production of copolymers with higher comonomer content than Ziegler–Natta catalysts and enable the synthesis of a variety of other materials, such as homopolymers and copolymers of cycloolefins, syndiotactic polystyrene, ethylene/styrene copolymers, etc. Since the 1990s, significant interest has arisen in catalysts based on well-defined transition metal complexes with non-cyclopentadienyl type ligands. Those catalysts with ligands containing donor heteroatoms, such as oxygen, nitrogen, sulfur, etc., termed “post-metallocene catalysts”, enable fine-tuning of chain microstructure of polymers by ligand design [7,8]. The presence of many donor atoms in a ligand usually has a good stabilizing effect on active sites. Moreover, the ligand structure is decisive for the geometry of the complex to be formed, for metal coordination number, and for the formal oxidation state of the transition metal [9].

The catalytic polymerization of ethylene leads to high density polyethylene (HDPE), which is a thermoplastic polymer with high softening point and high crystallinity, and has very good chemical resistance [10]. Its properties are strongly affected by molecular weight, and higher molecular weights are advantageous inter alia for mechanical properties of polyethylene. Classifications of PE materials depending on their molecular weights are presented in Table 1 [11,12]. Molecular weight distribution (MWD) of polyethylene is another factor which influences the properties of polyethylene. For example, broad MWD polyethylene offers good impact resistance and good processability, while narrow MWD polyethylene will have greater toughness at low temperatures and higher resistance to environmental stress-cracking [13,14].

Among the commercially available various grades of polyethylene (ref. to Table 2 and Table 3), of great importance are polyethylenes which, from the viewpoint of their chemical structures, are copolymers of ethylene with other monomers, mostly with 1-olefins. They are usually statistical copolymers differing in the amounts of comonomer incorporated and chemical compositions distributions. That group covers: linear low density polyethylene (LLDPE and produced by metallocene catalysts mLLDPE), very low density polyethylene (VLDPE) and ultra-low density polyethylene (ULDPE). Ethylene and 1-olefin copolymers are commercially produced with the use of Ziegler–Natta catalysts and metallocene catalysts including constrained geometry catalysts. Multiblock copolymers of ethylene and 1-olefins, called olefin block copolymers (OBC), are also commercially available, and are synthesized with the use of post-metallocene catalysts and a new coordination polymerization method, i.e., Chain Schuttling Polymerization (CSP) [7,16]. The amount of 1-olefin incorporated into the copolymer, as well as the microstructure of the copolymer, influences the product density and the physicochemical properties which are determining factors for its final application.

The properties of polyethylene can be modified, not only by introducing 1-olefin units, but also by copolymerization of ethylene with other compounds like norbornene, styrene, or polar monomers. The ethylene/norbornene copolymers, called cyclic olefin copolymers (COC), are successfully produced commercially and show high transparency, high humidity and thermal-resistance, and high water-vapor barrier properties. They are resistant to polar solvents and chemicals, too [17,18]. The introduction of styrene units into the polyethylene macromolecules causes changes in the viscoelastic behavior and thermo-mechanical properties of HDPE [19]. Functionalization of polyethylene through copolymerization of ethylene with polar comonomers is advantageous for the produced material from the viewpoint of its adhesion, toughness, printability/paintability, and rheological properties [20]. The copolymers of ethylene with polar comonomers or with styrene offer improved miscibility with other polymers [19,20]. It should be noted, however, that catalytic copolymerization of ethylene with polar monomers, for example, α,ω-alkenols, or styrene and its derivatives, faces some problems which result from the nature of those comonomers. Early transition metal catalysts have a tendency to form stable σ-complexes with the polar group of comonomer due to high oxophilicity of metal centers and, as a consequence, they can be easily poisoned [21]. Copolymerization of ethylene and styrenic monomers, on the other hand, often in addition to copolymer, yields homopolymer as contaminants. However, in the late 90s, production of ethylene/styrene copolymers, called pseudorandom interpolymers, was commercialized with the use of type CGC catalysts [22].

## 2. Copolymerization of Ethylene with Vinyl Monomers

### 2.1. Copolymerization of Ethylene with 1-Olefins

Copolymerization of ethylene with 1-olefins leads to structural changes in macromolecules of linear polyethylene, i.e., incorporated 1-olefins produce short branches the lengths of which are dependent on the type of the higher 1-olefin used. Such modifications cause changes in the crystallinity and density of polyethylene, and other physical properties, e.g., melting point, optical properties, as well as mechanical properties [25]. The ethylene/1-olefin copolymers show good strength, toughness, and sealing properties. Moreover, they offer advantageous optical properties which make them widely applicable in the packaging industry [24]. In addition to type of 1-olefin used, the properties of those materials depend on the amount of comonomer incorporated, as well as its distribution along the copolymer chains (inter- and intramolecular). The microstructure of copolymers, and other characteristics, like molecular weight and its distribution, are strictly related to the type of catalyst used for copolymerization. The metallocene catalysts make it possible to produce copolymers with higher degrees of comonomer incorporation, with more uniform comonomer distribution, and with narrower molecular weight distribution compared to the copolymers obtained with the use of Ziegler–Natta heterogeneous catalysts [26,28]. That results from existence of single site in metallocene catalysts and multiple active sites in the Ziegler–Natta catalysts with different reactivities toward the monomers, and from different accessibility of catalytic active sites for the comonomer molecules [26,28]. In addition to statistical ethylene/1-olefin copolymers, post-metallocene catalysts offer copolymers with new architecture, block copolymers, and multiblock copolymers. It became possible due to innovation in living polymerization of olefins and development of chain shuttling polymerization.

#### 2.1.1. Complexes of Group 4 Transition Metals

The post-metallocene catalysts, which are based on the complexes of group 4 transition metals, have been extensively examined in copolymerization of ethylene with 1-olefins. It is not possible to present the catalytic performance of all the groups of the investigated complexes within this short review. Hence, the survey is limited only to the most representative catalysts producing copolymers with different macromolecular structures. 

**Figure 1:**
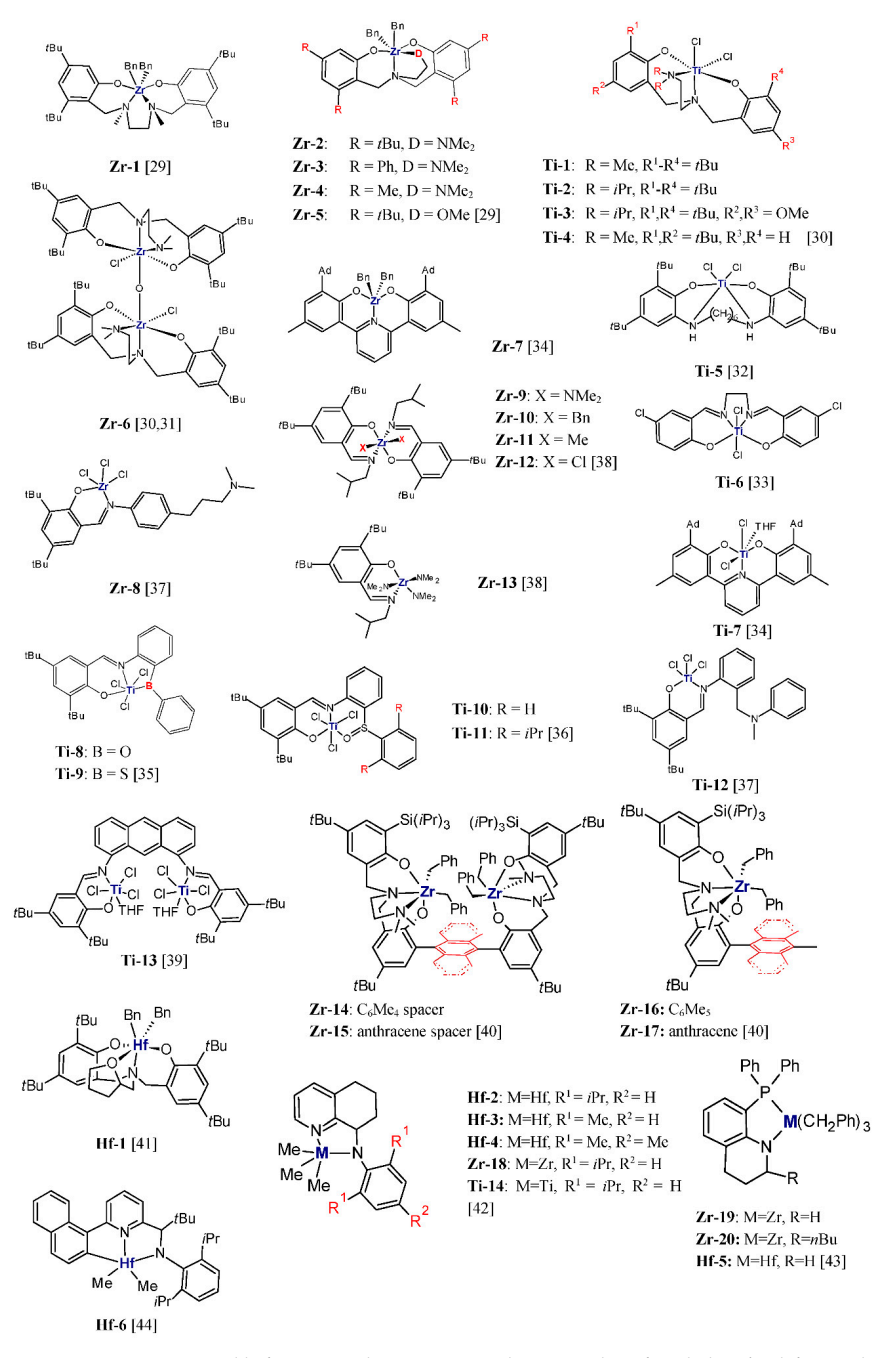
Titanium, zirconium and hafnium complexes investigated as precatalysts for ethylene/1-olefin copolymerization [29,30,31,32,33,34,35,36,37,38,39,40,41,42,43,44].

The titanium and zirconium complexes with tetradentate ligands make one of the groups most often used in olefin copolymerization. S.E. Reybuck et al. [29] compared the effects of the symmetry of complexes, steric bulk of the substituent at the aryl ring, and the type of activator on the ethylene/1-hexene copolymerization behavior of zirconium diamino-bis(phenolate) complexes **Zr-1–Zr-5** (Figure 1). The complexes with *C_s_* symmetry (**Zr-2**–**Zr-5**), i.e., those with the amino-bis(phenolate) ligands with the pendant donor group, were demonstrated to be more active and to offer a better ability to incorporate comonomer than those with *C_2_* symmetry (**Zr-1**). The copolymer produced by **Zr-2**/MMAO (MMAO- modified methylaluminoxane) at X_E_/X_H_ = 0.11 (mol/mol) contained 44.9 mol% 1-hexene, while the copolymer synthesized with **Zr-1**/MMAO at the same feed ratio of comonomers contained only 8.7 mol% of 1-hexene. The symmetry of the complex also affected the distribution of comonomer. The product of the reactivity ratios for copolymerization with **Zr-1** was higher than 1 (r_e_r_h_ = 2.6) and for copolymerization with **Zr-2** below 1 (r_e_r_h_ = 0.18), which suggested the presence of blocky and slightly alternating sequences of the comonomer units, respectively. It was also shown that the 1-hexene incorporation ability of the *C_s_* symmetric complexes increases in the order of decreasing steric bulk at the 2,4-aryl positions. However, bulky substituents like *tert*-butyl groups (**Zr-2**) or phenyl groups (**Zr-3**) were found to improve the complex activity at the same time. The copolymerization behavior of the complexes was also sensitive to the nature of the activator. Copolymers produced by *C_s_*-symmetric complexes activated with MMAO had higher 1-hexene incorporation by about 10 mol% than those synthesized with borates as activators. All the copolymers synthesized with the use of **Zr-1–Zr-5** complexes showed monomodal distribution and narrow molecular weight distribution (M_w_/M_n_ = 1.3–1.9), which is indicative for homogeneity of active sites in the catalysts [29].

The dimeric diamine-bisphenolate complex **Zr-6** activated with Al(*i*Bu)_3_/Ph_3_CB(C_6_F_5_)_4_ produced copolymers with very high 1-octene incorporation, up to ≈20 mol% at 0.70 mol/L of comonomer in the feed, with moderate molecular weight and dispersity (M_w_ = 14,000–42,000 g/mol, M_w_/M_n_ = 2.1–3.2). In addition, the activating effect of the comonomer on the copolymerization yield was observed independently on the comonomer concentration. Its activity was over four times higher in ethylene/1-octene copolymerization than in ethylene homopolymerization [30]. In contrast, the titanium complexes **Ti-1**–**Ti-4** bearing the same type of tetradentate ligand in conjunction with Al(*i*Bu)_3_/Ph_3_CB(C_6_F_5_)_4_ exhibited the negative comonomer effect and lower activity than the zirconium catalyst. The comonomer incorporation ability for these catalysts was also lower than for **Zr-6** and it was strictly dependent on the ligand structure. The complexes **Ti-1** and **Ti-4** with the NMe_2_ donor group gave copolymers with 9.0 and 12.0 mol% of 1-octene at the comonomer concentration equal to 0.70 mol/L. Interestingly, **Ti-4** produced the ethylene/1-octene copolymer, not only with a high incorporation level, but at the same time with the very high molecular weight and very narrow molecular weight distribution (M_w_ = 755,000 g/mol, M_w_/M_n_ = 1.7). The introduction of sterically bulkier N(*i*Pr)_2_ pendant donor group in the complexes **Ti-2** and **Ti-3** resulted in lower 1-octene contents: 5.1 mol% and 2.8 mol%, respectively [30].

Further studies [31] showed that the presence of AlMe_3_ in the feed during the copolymerization catalyzed by **Zr-6**/Al(*i*Bu)_3_/Ph_3_CB(C_6_F_5_)_4_ lowered the catalyst activity and greatly increased the amount of comonomer content in the copolymer from about 10 to approximately 40 mol% for 1-octene and 31 mol% for 1-hexene when the AlMe_3_/Zr molar ratio was equal to 100/1. The increase in the AlMe_3_/Zr molar ratio to 150:1 resulted in the production of ethylene/1-octene copolymer with the comonomer content of 65 mol%. The presence of AlMe_3_ significantly affected the microstructure of the produced copolymers which possessed both blocky and alternating sequences, and decreased the molecular weight of the produced copolymers. However, other alkylaluminium compounds, AlEt_3_ and Al(*i*Bu)_3_, changed molecular weight of copolymers but did not cause any important changes in the catalytic activity, composition, or microstructure of the copolymerization products. Hence, only AlMe_3_ interacts with the active sites to modify their catalytic properties [31].

Titanium catalyst **Ti-5**/MAO (MAO-methylaluminoxane), based on a complex with tetradentate salan type ligand, was investigated in ethylene/1-octene copolymerization as well. At the comonomer concentration of 0.73 mol/L, it gave the copolymer with moderate incorporation (3.7 mol%) and its activity was low (3.7 kg/(mol_Ti_·h)) [32]. Similarly, low activity and moderate incorporation ability were exhibited in ethylene/1-octene copolymerization by the titanium catalyst **Ti-6**/MAO, which was based on the tetradentate salen type ligand [33]. At the highest investigated comonomer concentration, 1.340 mol/L, the copolymer with 5.5 mol% of 1-octene was obtained and the catalytic activity was 14.7 kg/(mol_Ti_·0.5 h). The copolymers produced by **Ti-6** were characterized by very high molecular weights (M_w_ = 1023 × 10^3^–1545 × 10^3^ g/mol) and very broad molecular weight distributions.

Among the large number of group 4 post-metallocene catalysts reported for ethylene copolymerization, are those based on complexes with tridentate ligands. That group covers **Ti-7** and **Zr-7** complexes bearing the bisphenolate ligand with the pyridinediyl linker which were tested in ethylene/1-octene copolymerization [34]. Both complexes in conjunction with MAO turned out ineffective in that process. The titanium catalyst exhibited very low, 3 kg/(mol_Ti_·h), activity and **Zr-7**/MAO gave the copolymer which contained only 0.5 mol% of comonomer, despite low ethylene pressure (1 atm) and high concentration of 1-olefin (~50% vol.) in the feed. In contrast, the titanium complexes **Ti-8–Ti-10** bearing the tridentate monoanionic [ONX] (X = O, S) ligands, after activation with MMAO, showed much better capability to copolymerize ethylene and 1-hexene [35,36]. The complex **Ti-9** with S in the sidearm was both highly active (387 kg/[mol_Ti_·h·atm]) and gave a copolymer with the very high 1-hexene content (30 mol%). The corresponding complex with an O-donor as the sidearm (**Ti-8**) under the same reaction conditions produced copolymers with lower activity and comonomer incorporation equal to 23 kg/(mol_Ti_·h·atm) and 14.1 mol%, respectively. The excellent copolymerization behavior showed the complex **Ti-10** with sulfoxide group [36] which produced copolymer with incorporation levels as high as 38.8 mol% and the activity of 320 kg/(mol_Ti_·h·atm). These results show that pendant donor group in phenoxy-imine ligand can easily tune the activity of catalyst and its capability for copolymerization. Further investigations showed that substituent on the sidearm also influenced strongly the copolymerization behavior of the complexes. In contrast to **Ti-10**, complex **Ti-11,** due to the steric effects of the isopropyl group, produced copolymer with very low comonomer content (0.6 mol%) [36]. Those results demonstrate that the catalytic properties of **Ti-8**–**Ti-11** can be well correlated with the steric and electronic properties of the side arm donor group [35].

When the zirconium **Zr-8** and titanium **Ti-12** complexes bearing potentially tridentate phenoxy-imine ligands were employed in ethylene/1-octene copolymerization in the presence of MMAO, their activities were very high (≈12,300 kg/[mol_Zr_·h]) and moderate (≈450 kg/[mol_Ti_·h]). However, their comonomer incorporation abilities were very low (up to 0.1 mol%) and low (up to 1.5 mol%), respectively [37]. When activated with Al(*i*Bu)_3_/Ph_3_CB(C_6_F_5_)_4_, these complexes were able to produce copolymers with higher comonomer contents (1.6 and 3.8 mol%, respectively), yet their activities were much lower. Interestingly, the produced copolymers showed broad chemical composition distributions and very broad molecular weight distributions, as well as high molecular weights irrespective of the comonomer concentration in the feed. At the 1-octene concentration of 0.58 mol/L, **Zr-8** produced copolymers with M_w_ = 440,000 g/mol and M_w_/M_n_ = 116 (trimodal distribution), whereas **Ti-12** gave the product with M_w_ = 690,000 g/mol and M_w_/M_n_ = 26.5 (bimodal distribution) [37].

Y. Gao et al. [38] studied ethylene/1-octene copolymerization with the use of phenoxy-imine complexes of zirconium (**Zr-9**–**Zr-12**). In contrast to other studies, they did not focus on modification of ancillary ligand structure, but on changing the type of monodentate σ-ligands. The synthesized complexes had chloride, methyl, benzyl, and dimethylamine as labile ligands. Such modification of precatalyst **Zr-9**–**Zr-12** was found to have considerable effect on their copolymerization behavior. Poly(ethylene-*co*-1-octene) produced by **Zr-9**/AlMe_3_/Ph_3_CB(C_6_F_5_)_4_ contained 7.2 mol% of comonomer. Under the same conditions (5 mL of 1-octene, 4 atm of ethylene, 40 °C, Zr/AlMe_3_ = 40), complexes with benzyl and methyl σ-ligands, **Zr-10** and **Zr-11**, gave copolymers with much lower 1-olefin incorporation, ≤1 mol%. Then, the complex **Zr-12** with the chloride labile ligand turned out inactive in copolymerization of ethylene and 1-octene. The comparison of the copolymerization results for the bis-FI and mono-FI complexes **Zr-9** and **Zr-13** revealed that both complexes are capable of producing copolymers with high comonomer incorporation, with 6.3 and 7.2 mol% of 1-octene, respectively, albeit with different activities. The activity of **Zr-9** with two phenoxy-imine ligands was 2846 kg/(mol·h·atm) while that of **Zr-13** was equal to 1812 kg. These two complexes behaved even more similarly at **Zr**/AlMe_3_ = 120 which could indicate that both those complexes form similar or identical cationic mono-FI-Zr catalytic species [38].

Recently, dinuclear titanium complex bearing anthracene-briged bisphenoxyimine ligand (**Ti-13**) activated with MAO was investigated as a precatalyst for ethylene/1-hexene copolymerization [39]. However, it was found to exhibit rather low activity and comonomer incorporation ability (303 kg/(mol_Ti_·h), 1.8 mol%). In contrast, *C_2_* symetric dinuclear zirconium catalysts supported by bisphenolate ligands with bulky Si(*i*Pr)_3_ substituent, **Zr-14** and **Zr-15**, in conjunction with MAO showed high activity and produced copolymers with good 1-hexene incorporation. At 3 bar of ethylene and 20 mL of comonomer in the feed, their activity and 1-hexene incorporation were equal to 2000 kg/(mol_Zr_·h), 22 mol% and 1900 kg/(mol_Zr_·h), 30 mol%, respectively. Their monometallic analogues, **Zr-16** and **Zr-17**, showed slightly lower incorporation ability (19 mol% and 28 mol%) [40]. It was also revealed that **Zr-14–Zr-17** incorporate the longer chain 1-olefin, 1-tetradecene, at lower level (3 mol%–10 mol%). 

Ethylene and 1-hexene were also copolymerized by hafnium-amine bis(phenolate) complex with tetrahydrofuran pendant arm (**Hf-1**), activated by B(C_6_F_5_) [41]. The catalyst was able to produce copolymers over a wide range of 1-hexene incorporation, from 25 mol% to 85 mol%, narrow dispersity, and M_n_ = 5600–47500 g/mol by changing comonomer concentration from 0.1 to 2.5 mol/L and reaction time from 10 to 90 min. However, its activity was modest (5.4–41.6 g/(mmol_Hf_·h)). Amido-trihydroquinoline hafnium complexes **Hf-2, Hf-3** and **Hf-4** with Ph_3_CB(C_6_F_5_)_4_ as the activator showed both high activity and ability to produce copolymers with high 1-octene content and high molecular weights (224,000–1220,000 g/mol) at high temperature (100 °C). At 1-octene concentration equal to 1 mol/L their activity decreased in the order **Hf-2**, 16,200 kg/(mol_Hf_·h) >> **Hf-4**, 2520 kg/(mol_Hf_·h) **> Hf-3**, 2340 kg/(mol_Hf_·h) and incorporation ability was changed as follows: **Hf-2** (15.6 mol%) > **Hf-3** (7.8 mol%) > **Hf-4** (4.8 mol%), indicating significant substituent steric effect on catalytic properties of hafnium catalysts [42]. Zirconium complex **Zr-18** bearing the same ligand as complex **Hf-2** was also highly efficient in ethylene/1-octene copolymerization (13,500 kg/(mol_Zr_·h), 12.4 mol%) but the exchange of Hf by Ti (**Ti-14**) led to catalyst with lower activity and low incorporation ability (2160 kg/(mol_Ti_·h), 1.7 mol%) [42].

Phosphine–amido hafnium and zirconium complexes bearing various substituents at the 2-position of the tetrahydroquinoline framework (R = H, Me, *i*Pr, *n*Bu) were evaluated in ethylene/1-octene copolymerization with [HNMe(C_18_H_37_)_2_][B(C_6_F_5_)_4_] as activator and under the following conditions: 1 mol/L of 1-octene, 30 bar of ethylene, 100 °C (initial reaction temperature), and 3 min [43]. Moderate 1-octene incorporation (7.7 mol%) showed zirconium complex **Zr-19**, however its Hf counterpart (**Hf-5**) and the other Zr and Hf complexes exhibited low comonomer incorporation (2.1 mol%–3.6 mol%). Activities of complexes fell in the range of 7000–29,000 kg/(mol_M_·h), with the highest and lowest activity shown by **Zr-20** and **Hf-5**, respectively. 

One of the interesting classes of postmetallocene catalysts are catalysts based on pyridylamido hafnium complexes which were shown to have high incorporation ability in ethylene/1-olefin copolymerization at high temperature [44,45,46]. *C*_1_-symmetric *tert*-butyl substituted pyridylamido hafnium complex **Hf-6** activated with Ph_3_CB(C_6_F_5_)_4_/Al(*i*Bu)_3_ was able to incorporate 6.0 mol% of 1-octene and 8.7 mol% of 1-hexene in ethylene/1-olefin copolymerization carried out with the addition of 0.1 mol of comonomer, at 80 °C for 10 min under 10 atm of ethylene. The produced copolymers had broad MWD with bimodal distribution and high molecular weight equal to 262,000 g/mol and 931,000 g/mol (copolymer with 1-octene and 1-hexene, respectively) [44]. Selected results of ethylene/1-olefin copolymerization with group 4 metal complexes are displayed in Table 4.

**Figure 2:**
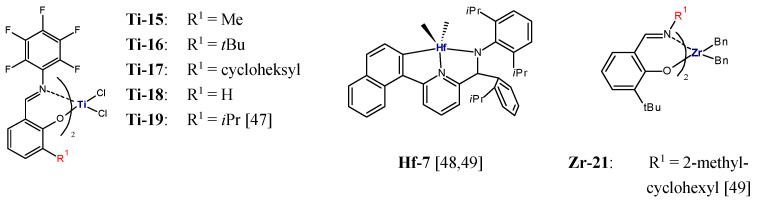
Fluorinated bis(phenoxy-imine) titanium complexes as well as bis(phenoxy-imine) zirconium and pyridylamide hafnium complexes used for synthesis of multiblock copolymers [47,48,49].

Fluorinated bis(phenoxy-imine)titanium complexes **Ti-15**, **Ti-16**, **Ti-17**, **Ti-18**, and **Ti-19** (Figure 2) activated by MAO were also applied in ethylene/1-olefin copolymerization (1-olefin = 1-hexene, 1-octene, 1-decene) [47]. All the catalysts were shown to offer high productivity. However, they had significantly different abilities to incorporate 1-olefins; this was dependent on the steric hindrance of the *ortho*-substituent. Reduction in the bulkiness of the substituent R enhanced reactivity of the catalysts towards higher 1-olefins. **Ti-18** (R^1^ = H) displayed the highest 1-hexene incorporation ability (22.6 mol%), whereas **Ti-16** (R^1^ = *t*Bu) showed the lowest efficiency (3.2 mol%). Moreover, the produced copolymers had very narrow molecular weight distributions (M_w_/M_n_ = 1.07–1.22) which indicated that the living ethylene/1-olefin copolymerization took place in the presence of those catalysts. 

The living polymerization which occurs with rapid initiation and negligible chain termination or chain transfer makes it possible to synthesize copolymers with well-defined blocks [50]. Most of the catalysts displayed the living olefin polymerization characteristics at low temperatures which resulted in low activity and insufficient molecular weights of the polymers [51,52]. Bis(phenoxy-imine) titanium catalysts which incorporate fluorine atom(s) ortho to the imine-nitrogen can catalyze the living (co)polymerization of olefins at ambient and elevated temperatures [51]. In the presence of **Ti-15**/MAO, a number of block copolymers of type PE-*block*-poly(ethylene-*co*-1-hexene) were synthesized using the sequential addition polymerization procedure. Linear polyethylene macromolecules (M_n_ = 38,300 g/mol, M_w_/M_n_ = 1.11) were obtained initially and then a mixture of ethylene and 1-hexene monomers was fed to obtain another block. The produced block copolymers contained from 3.3 mol% to 15.0 mol% of 1-hexene and were characterized by high molecular weights (M_n_ = 61,500–121,000 g/mol), narrow MWD (M_w_/M_n_ = 1.21–1.31), and melting points from 117 °C to 130 °C [47]. The block copolymers of that type were shown to possess a good combination of extensibility and toughness compared to the corresponding random copolymers.

**Figure 3:**
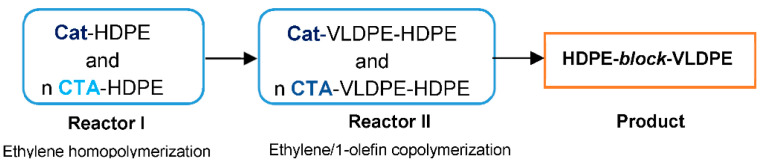
Synthesis of HDPE-*block*-VLDPE copolymer using coordinative chain transfer copolymerization and two reactors. Adopted from [48].

Further, the coordinative chain transfer copolymerization, CCTcoP, of ethylene with 1-olefins was developed. It was aimed at reducing the amounts of transition metal catalysts needed and controlling the molecular weight of polymers. In this approach, the growing polymer chain is capable of transferring from the catalyst to the chain transfer agent (CTA), which is a dormant species in the course of copolymerization [53]. P.D. Hustad et al. reported the method for the synthesis of block copolymers in the continuous process using CCT*co*P, with **Hf-7**/MAO as a catalyst (Figure 2) and ZnEt_2_ as CTA [48]. Two reactors were employed in the process (Figure 3). Ethylene homopolymerization took place in reactor I, while copolymerization of ethylene and 1-olefin was carried out in reactor II. When the HDPE-*block*-VLDPE copolymers obtained with CTA were compared with the products synthesized with no CTA (physical blend obtained in a dual-reactor), it was found that the copolymer produced with the use of CTA showed higher molecular weight, narrower MWD (M_n_ = 44.5 kg/mol, M_w_/M_n_ = 1.67), and somewhat lower melting point (T_m_ = 122 °C) than the product obtained with no use of CTA (M_n_ = 25.9 kg/mol, M_w_/M_n_ = 4.42, T_m_ = 126 °C). In that type of copolymerization, both the block composition and the comonomer content in each block can be easily tailored by changing the production rate in reactor I and reactor II, and by feed composition, respectively [48].

Block copolymers with novel architecture, showing excellent elastomeric properties, were introduced by Arriola et al. [49]. Linear multiblock ethylene/1-octene copolymers, with sequential crystallizable segments (low 1-octene content) and amorphous segments (high 1-octene content) were synthesized via chain shuttling polymerization. That type of polymerization involves two catalysts with different comonomer incorporation abilities and a chain shuttling agent (CSA) which is able to pass the growing polymer chain between catalytic sites so that different parts of a single polymer molecule grow on different catalysts [49,53]. The pyridylamide complex **Hf-7**, with the high ability to incorporate 1-octene, and the bis(phenoxy-imine) complex **Zr-21** (Figure 2), with low incorporation ability, combined with ZnEt_2_ as a CSA, made it possible to obtained multiblock copolymers which were characterized by very low densities (0.879–0.883 g/cm^3^) and high melting points (T_m_ = 120–124 °C) at the same time. Moreover, unlike the copolymer prepared without ZnEt_2_, which was bimodal with M_w_/M_n_ = 13.8, they show monomodal and narrow distributions of molecular weights (M_w_/M_n_ = 1.98–3.22) [49].

#### 2.1.2. Complexes of Vanadium

The vanadium catalysts for the olefin polymerization have been known since the 1950s and such classical Ziegler–Natta ones possess some interesting properties, which made them applicable in the commercial production of ethylene/propylene/diene and ethylene/cyclic olefins copolymers [54]. Although metallocene-type vanadium catalysts did not attract much attention of the researchers, the vanadium catalysts based on complexes with multidentate ligands started drawing considerably more and more interest as their catalytic performance, including thermal stability, can be modified by the use of specific multidentate ligands. The detailed review of the vanadium catalysts, which were employed in the olefin polymerization processes within the last decade, was published by A. M. F. Phillips et al. in Coordination Chemistry Reviews in 2020 [55], and earlier works were summarized by S. Gambarotta in Coordination Chemistry Reviews in 2003 [56]. Here, we summarize some interesting developments of the vanadium complexes for ethylene/1-olefin copolymerization.

**Figure 4:**
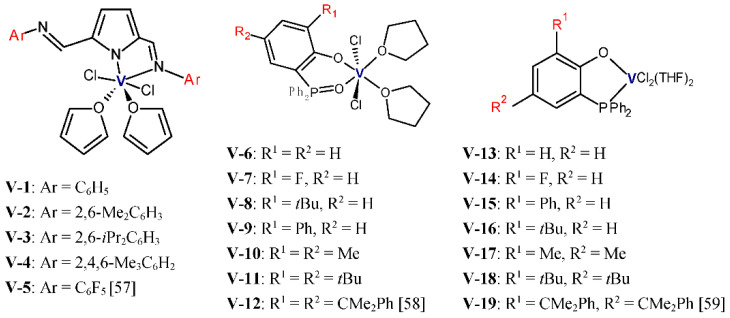
Vanadium complexes with bidentate ligands investigated as precatalysts for ethylene/1-olefin copolymerization [57,58,59].

Y.S. Li et al. studied the performance of vanadium(III) complexes (Figure 4) bearing bidentate ligands with [O,P], [N,N], and [O,O] donor atoms in ethylene/1-hexene copolymerization [57,58,59]. Et_2_AlCl and Cl_3_CCOOEt (ETA) were used as the activator and the reactivator, respectively. The copolymerizations were carried out at 1 atm of ethylene, 1-hexene concentration equal to 0.2 mol/L, and at 25 °C for 5 or 10 min. The complexes **V-1**–**V-5** showed high activities, 4.08–7.44 kg/(mmol_V_·h), and they produced copolymers with comonomer content between 2.22–4.20 mol% and with very narrow molecular weight distribution (M_w_/M_n_ ≤ 1.9). For the complexes with electron donating imino groups, the 1-hexene incorporation ability was shown to increase in line with the decreasing steric hindrance at the 2,6-aryl positions (**V-3** < **V-2**, **V-4** < **V-1**). The presence of the electron withdrawing group in the complex decreased the 1-hexene incorporation (**V-1** > **V-5**). In addition, the properties of the resultant copolymers can be controlled over a wide range by changing the reaction parameters. At 75 °C and at 1-hexene initial concentration of 0.6 mol/L, the **V-3**/Et_2_AlCl system was found to produce the copolymer with 1-hexene incorporation as high as 16 mol% [57]. In general, the complexes **V-6**–**V-12** and **V-13**–**V-19** exhibited good activities and incorporation ability in the range of 1.08-5.34 kg/(mmol_V_·h), 2.34–5.21 mol% [58], 0.84–4.56 kg/(mmol_V_·h), and 2.33–3.75 mol% [59], respectively. Nevertheless, their copolymerization behavior is influenced by the nature of substituents in the ligands. The increasing steric hindrance on the aryloxy moiety of the phenoxy-phosphine ligand was shown to improve the catalytic activity (**V-13** < **V-15** < **V-16**, and **V-17** < **V-18**). However, the increased steric bulk of the ligand in **V-19** slightly lowered the catalytic activity for copolymerization (**V-18** > **V-19**). In addition, the electron-withdrawing effect of the F-substituted phenoxy-phosphine ligand influenced the catalytic performance unfavorably [59]. Moreover, the steric hindrance of the ligand and the electron-withdrawing effect of the ligand was also unfavorable for 1-hexene insertion.

**Figure 5:**
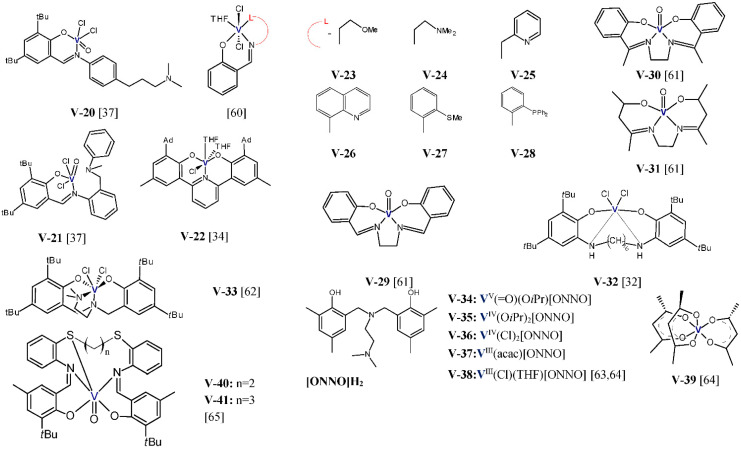
Vanadium complexes investigated as precatalysts for ethylene/1-olefin copolymerization [32,34,37,60,61,62,63,64,65].

The vanadium complexes **V-20** and **V-21** (Figure 5), activated either with EtAlCl_2_ or with Et_2_AlCl, were also examined in ethylene/1-octene copolymerization [37]. The copolymerization tests were carried out in the presence of ETA at 60 °C, for 20 min, under 5 bar ethylene, and in the presence of 2, 5, and 6.5 mL 1-octene. Both these complexes in conjunction with EtAlCl_2_ exhibited high catalytic activities (66,400–10,400 kg/(mol_V_·h)) and, taking into account the high pressure of ethylene and low comonomer concentration, they offered acceptable incorporation ability (1.3–3.6 mol%). The produced copolymers were characterized by high molecular weights and low MWD (M_w_ = 380,000–120,000 g/mol, M_w_/M_n_ ≤ 2). When the activator was changed for Et_2_AlCl, both complexes were less active and they produced copolymers with lower comonomer contents and high dispersity (M_w_/M_n_ = 6.4–7.9).

S.R. Golisz [34] reported the use of the vanadium complex **V-22** with the tridentate bis(phenolate) ligand with the pyridinediyl linker, which was analogous to the complexes **Ti-7** and **Zr-7**, in copolymerization of ethylene and 1-octene. **V-22**/MAO exhibited low activity (50 kg/[mol_v_⋅h]) and moderate incorporation ability. The copolymer obtained at high 1-octene/ethylene ratio contained 6.6 mol% of comonomer; still, it was much higher than for the use of group 4 catalysts under the same conditions. J-Q. Wu et al. [60] studied the performance of the vanadium(III) complexes **V-23**–**V-28** bearing tridentate phenoxy-imine ligands with different pendant donor groups, in copolymerization of ethylene with 1-hexene. At the same copolymerization conditions: 0.27 mol/L 1-hexene, 1 bar ethylene, at 25 °C, with Et_2_AlCl as an activator, and ETA as a reactivator, the studied complexes were characterized by different activities, from 0.3 kg/(mmol_V_·h·bar) up to 3.61 kg/(mmol_V_·h·bar), with the figures changing as follows: **V-23** < **V-27** < **V-24** < **V-26** < **V-28** < **V-25**. Hence, the results were dependent both on the pendant donor and on the electronic effect of the backbone of the ligands. All the catalysts produced copolymers with moderate comonomer contents, 2.3–3.2 mol%, with high molecular weights, 41,800–93,800 g/mol, and with unimodal molecular weight distributions (M_w_/M_n_ = 1.9–2.9). The most active complex, **V-25**, produced the copolymer with 3 mol% of 1-hexene and its content in the copolymer increased to 12.9 mol% when the comonomer concentration was raised to 1.35 mol/L. Moreover, enhanced catalytic activity was observed for the catalyst **V-25** with the increasing polymerization temperature up to 50 °C, which is indicative for its good thermal stability.

The oxovanadium(V) complexes (**V-29**–**V-31**) with tetradentate Schiff base ligands were also used in copolymerization of ethylene with 1-olefins as precatalysts. Those complexes, when activated with EtAlCl_2_, make it possible to produce poly(ethylene-*co*-1-octene) with the comonomer content from 5.26 to 6.25 mol% and melting points ~112 °C, at the comonomer concentration of 0.40 mol/L. The copolymer with the highest 1-octene content was obtained with the use of **V-29** and it was found to contain isolated comonomer units only [61]. The discussed complexes offered low catalytic activities in copolymerization, from 2.6 kg/(mol_V_·h) to 18.1 kg/(mol_V_·h), and it grew up in the following line: **V-29** < **V-30** < **V-31**. Higher activities in ethylene/1-octene copolymerization, from 1193.5 to 197.6 kg/(mol_V_·h), at the comonomer concentrations changing from 0.20 to 0.73 mol/L, were exhibited by the vanadium catalyst **V-32**/EtAlCl_2_ which was based on the complex with the tetradentate salan-type ligand. However, no neat copolymer was formed at higher comonomer concentrations, but the mixtures of products were obtained [32].

The copolymerization process of ethylene with 1-octene was also explored for three different comonomer concentrations: 0.19, 0.38, and 0.70 mol/L, with the use of the vanadium(IV) complex **V-33** with the diamino-bis(phenolate) ligand, which was activated by EtAlCl_2_ [62]. Catalyst activity fell within 350–120 kg/(mol_V_·h) and it decreased for the increasing comonomer concentrations. The comonomer incorporation was equal to 4.1 mol% at the lowest comonomer concentration and it increased to 9.1 mol% for 0.70 mol/L. At this comonomer concentration, however, a by-product was formed as a colorless oily liquid in addition to the solid copolymer. It should also be noted that the activator considerably affected the copolymerization process. When the activator was changed for Al(*i*Bu)_3_/Ph_3_CB(C_6_F_5_)_4_, some lowering of the catalytic activity and considerably lower 1-octene incorporation was observed. The obtained copolymer showed a high molecular weight (M_w_ ~ 1 × 10^6^ g/mol) and narrow MWD (M_w_/M_n_ = 1.4) [62]. 

Lorber et al. employed the vanadium(III-V) complexes **V-34**–**V-38** bearing similar diamine-bis(phenolate) ligands in the ethylene/1-hexene copolymerization reaction [63,64]. The copolymerizations were conducted in the presence of the EtAlCl_2_ activator, under 2 bars ethylene, and with 8 mmol 1-hexene. Although **V-38** did not produce any copolymer, the complexes **V-34**–**V-37** gave the products which contained 3.5–7.4 mol% comonomer, with the activity from 12 to 120 kg/(mol_V_·h). The V(III) complex **V-37** exhibited both the highest activity and incorporation ability. When the catalytic properties were compared for **V-37**/EtAlCl_2_ and for the classical-type system based on vanadium acetylacetonate, **V-39**/EtAlCl_2_, those catalysts were found to have similar comonomer incorporation abilities: they produced copolymers with 7.4 mol% and 7.5 mol% comonomer. However, the distinguishing feature of the copolymer synthesized with **V-39** was its very high dispersity (M_w_/M_n_ = 225) versus the copolymer produced with **V-37** (M_w_/M_n_ = 1.7). The catalytic performance of **V-37** in copolymerization of ethylene with 1-hexene and ethylene with 1-octene was additionally compared in [64]. It was found that, under the same conditions, the copolymers with higher amounts of comonomer units were created in copolymerization of ethylene with 1-octene (8.0–12.0 mol% vs. 7.4–10.6 mol%).

Vanadium complexes bearing ligands featuring sulfur, nitrogen, and oxygen donors were screened for the copolymerization of ethylene and 1-hexene in the presence of dimethylaluminum chloride as an activator and ETA as reactivator by Homden et al. [65]. Copolymerizations were performed at 25 °C for 30 min under 1 bar of ethylene and with different comonomer concentrations. It was found that **V-40** exhibited the highest incorporation ability (11.3 mol% at 4000 equv of 1-hexene) and the highest activity, 1190 kg/(mol_V_·h), showed **V-41.** Selected results of ethylene/1-olefin copolymerization with vanadium complexes were displayed in Table 5.

As results from the review of the literature reports on copolymerization of ethylene with 1-olefins show, it is hard to directly compare the performance of the complexes which are used in this process due to the different copolymerization conditions adopted for the experiments, and, in particular, ethylene pressure and 1-olefin concentration values. The studied complexes contained ligands with various denticity and with different donor atoms, and their catalytic performance in copolymerization may be significantly modified, not only by ligand backbone, but also by the type of substituents on the aromatic rings (the effects of their steric and electron donor properties need to be considered). Since the living polymerization catalysts and new coordination polymerization techniques were developed, the group 4 metal complexes made it possible to obtain block copolymers and multiblock copolymers which, so far, could not be produced with the use of the vanadium catalysts. It should also be noted that when the vanadium complexes and the titanium/zirconium complexes with the ligands of the same structure are used in copolymerization, the former usually exhibit higher comonomer incorporation ability and produce more uniform copolymers. However, it should also be stressed that when ethylene/1-octene copolymerization with vanadium complexes activated by EtAlCl_2_ is conducted with no reactivator, higher comonomer concentrations in the reaction medium may yield mixture of products (copolymerization in the presence of **V-32** and **V-33**). This is probably due to the simultaneous presence of various types of active sites formed by reduction of the initial vanadium compound.

### 2.2. Copolymerization of Ethylene with Styrene and Its Derivatives

The properties of polyethylene can be also modified by incorporating units which come from styrene or its derivatives. Depending on the amount of the incorporated styrene units, the products may be from semicrystalline to amorphous [19]. It is hard to copolymerize ethylene with styrene because their reactivity towards most of the catalysts is different, and because of the copolymers chains can be produced together with homopolymers [22,66]. It is for that reason as well as low activity and low styrene incorporation (usually below 1 mol%) that the Ziegler–Natta catalysts failed in the synthesis of ethylene/styrene copolymers [19]. The type CGC homogeneous catalysts turned out undoubtably better in the copolymerization of ethylene and styrene monomers [66,67,68,69,70]. Based on single-site, constrained-geometry catalysts, the INSITE technology was developed for the production of ethylene/styrene interpolymers which are described as “pseudo-random” ones, and contain typically up to 50 mol% of styrene [67,71]. Complexes with the monocyclopentadienyl ligands [72,73,74] and *ansa*-zirconocenes [75,76] are also used as precursors for ethylene/styrene copolymerization. Their use made it possible to obtain copolymers with various compositions, microstructures and properties, and namely copolymers with isolated styrene units, stereoregular Et-St alternating sequences or block copolymers with isotactic polystyrene blocks [75,76,77,78].

#### Complexes of Group 4 Transition Metals

**Figure 6:**
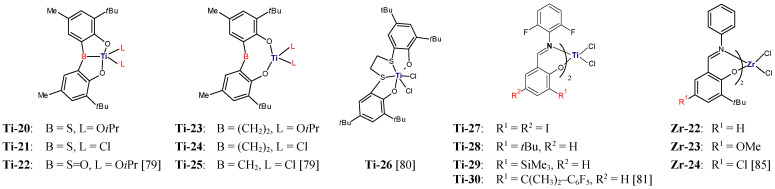
Titanium and zirconium complexes investigated as precatalysts for ethylene/styrene copolymerization [79,80,81,85].

Early transition metal complexes with chelating ligands have attracted a lot of attention as precatalyst in ethylene/1-olefin polymerization. However, there are not many reports of their use in ethylene/styrene copolymerization. That process employed inter alia titanium bis(phenolate) complexes **Ti**-**20**–**Ti**-**25** (Figure 6) activated with methylaluminoxane [79]. The type of the bridging unit between the phenolate moieties of the ligand was shown to be decisive for incorporation of styrene and catalytic activity. The complexes with the sulfur bridge were observed to be most active and to promote lowest styrene incorporation at the same time. For the reaction conducted at 60 °C and with the styrene/ethylene molar ratio of 5/1, the reported activity and the comonomer content in the copolymer were: 82 kg/(mol_Ti_·h·mol/L), 6.0 mol% (**Ti-21**); and 109 kg/(mol_Ti_·h·mol/L), 5.5 mol% (**Ti-20**), respectively. The activity of the complexes with the ethylene bridge amounted to 1 kg/(mol_Ti_·h·mol/L) and the comonomer incorporation level reached about 35.5 mol% under the same conditions. The sulfoxy-bridged complex, **Ti-22**, gave the styrene incorporation of 10 mol% with the activity of 10 kg/(mol_Ti_·h·mol/L), and the methylene-bridged complex, **Ti-25,** did not make it possible to incorporate any styrene at all. Such changes in catalytic activity of complexes indicate that the additional coordination of the bridging unit to the titanium center leads to the increased Lewis-acidity and consequently to the increased catalyst activity. On the other hand, the steric hindrance at the active center was also affected by the type of the bridging group and it was responsible for the decreased styrene incorporation. The detailed analysis demonstrated that **Ti-20**/MAO produce random poly(ethylene-*co*-styrene) with broad distribution of molecular weight and the comonomer incorporation [79].

The 1,4-dithiabutanediyl-linked bis(phenolato) titanium complex activated by methylaluminoxane, **Ti-26**/MAO produced exclusively ethylene/styrene copolymers with high activity, up to 1600 kg/(mol_Ti_·h) [80]. Synthesized copolymers had a broad range of styrene content, from 23 to 68 mol%, and their microstructures were dependent on their compositions. The copolymers with the styrene content of 40 mol% were pseudorandom, and the presence of styrene–styrene homo-sequences (SSS) and isolated ethylene units was observed for the copolymers with higher styrene contents [80]. Synthesis of PE-*block*-PS the diblock copolymers by sequential monomer addition in the presence of various phenoxyimine complexes (**Ti-27**–**Ti-30**), suitable for living olefin polymerization, i.e., with fluorine substituents, activated by MAO, was reported in [81]. In fact, a blend of the block copolymers and polyethylene was obtained and, at high styrene concentrations, atactic polystyrene was formed as a by-product. It was also found that copolymers synthesized with **Ti-27**/MAO possessed ultra-high molecular weights, with M_n_ = 540,000–850,000 g/mol for polyethylene blocks and with M_n_ = 40,000–90,000 g/mol for polystyrene blocks [81]. 

The copolymerization experiments involving ethylene and styrene derivatives were conducted principally with metallocene, CGC, or scandium catalysts [82,83,84]. However, the examples described below show that the replacement of styrene with its derivative may induce essential change in the copolymerization behavior of post-metallocene catalysts. The bis(phenoxy-imine) zirconium catalysts, **Zr-22**–**Zr-24**/MAO, were not able to produce ethylene/styrene copolymers, probably due to the dominant mechanism of the 2,1-insertion, which resulted in the formation of unreactive benzyls [85]. Replacement of styrene with *para-tert*-butylstyrene (TBS) made it possible to conduct the reaction and to produce the copolymer with substantial comonomer incorporation, from 4.9 to 11.3 mol% at a relatively low concentration of comonomer (0.7 mol/L). The copolymers were also characterized by narrow molecular weight distribution (M_w_/M_n_ = 1.86–2.84) and lack of sequential TBS units and TBS-E-TBS triads. Moreover, on moving from ethylene homopolymerization to ethylene/*para-tert*-butylstyrene copolymerization, only a small reduction in the catalyst activity was observed [85]. 

**Figure 7:**
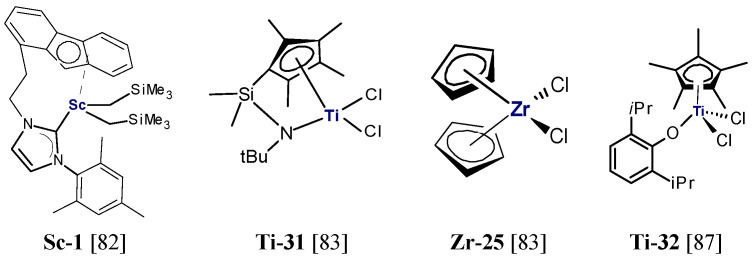
Complexes investigated as precatalysts for copolymerization of ethylene with alkyl derivatives of styrene or α,ω-alkenols [82,83,87].

Copolymerization of ethylene with styrene and its derivatives with electron-donating substituents at the para position, *para*-methylstyrene (MS), and TBS, was described in [86]. It was catalyzed by diamine-bis(phenolate) complexes of zirconium (**Zr-6**) and titanium (**Ti-1**) activated either by MMAO or by Al(*i*Bu)_3_/Ph_3_CB(C_6_F_5_)_4_. Copolymerization with **Zr-6**/MMAO was found to produce, exclusively, copolymers with the incorporation level of 2.2–4.6 mol%, whereas other catalytic systems yielded mixtures of copolymers and homopolymers. The catalytic activity of **Zr-6**/MMAO in ethylene/styrene copolymerization at 88.5 mmol comonomer and at 5 bar ethylene was equal to 613.6 kg/(mol_Zr_·h) and was higher than in ethylene homopolymerization (203.0 kg/[mol_Zr_·h]); it increased to 985.4 and 1027.2 kg/(mol_Zr_·h) after changing the comonomer to MS and TBS, respectively. This effect of a styrenic monomer on catalyst activity is consistent with previous findings observed for the catalysts based on **Sc-1**, **Ti-31** and **Zr-25** (Figure 7) [82,83]. Selected results of ethylene/styrene copolymerization were displayed in Table 6.

As far as we know, the post-metallocene catalysts based on complexes of group 5 transition metals were not examined in copolymerization of ethylene and styrenic monomers.

### 2.3. Copolymerization of Ethylene with α,ω-Alkenols 

Functionalized polyolefins can be synthesized in numerous ways. Polymer post-functionalization is one of the approaches used. That method requires harsh conditions and may lead to undesirable side reactions which eventually change the properties of the polymer in an unwanted manner [20]. The ring-opening metathesis polymerization of functionalized cyclic alkenes and acyclic diene metathesis polycondensation, both followed by hydrogenation, are other approaches for obtaining functionalized polyolefin. However, a multi-step fashion and special monomers needed for those methods are their main drawbacks [20]. An alternative method for the production of functionalized polyolefins is the free-radical copolymerization of olefins and polar vinyl monomers, which is widely applied in industry. This, however, does not provide satisfactory control of the composition and the microstructures of the produced copolymers. It is definitely easier to control the composition and the polar monomer distribution along the polymer backbone in the direct copolymerization of ethylene and polar comonomers promoted by organometallic catalysts. As polar monomers, acrylates, vinyl ethers, vinyl halides, unsaturated carboxylic acids, and esters, mostly methyl esters of those acids, α,ω-alkenols, and others, were used [88,89]. Although late transition-metal catalysts are able to incorporate the unprotected polar vinyl monomers, early transition-metal complexes require the protection of the polar groups to prevent partial or complete deactivation of the catalyst [20,88,89,90]. One of the groups of polar monomers are unsaturated alcohols with various spacers between the double bond and the functionality, like 5-heksen-1-ol (5H1O), 9-decen-1-ol (9D1O), and 10-undecen-1-ol (10U1O). In order to protect the catalytic active sites against coordination of polar groups and their deactivation, α,ω-alkenols are most frequently reacted with the organoaluminium compounds.

The use of Ziegler–Natta catalysts in the ethylene/α,ω-alkenols copolymerization, alike with other monomers, has limitations because of their low comonomer incorporation ability. More comprehensive and more effective attempts to functionalize polyethylene were carried out in the presence of metallocene catalytic systems. The research in this field was reviewed in detail by M. Atiqullah et al. [91]. Later, copolymerization of ethylene and 4-penten-1-ol as well as 9D1O was shown to be effective by using the half-titanocene catalyst (**Ti-32**) with the additional aryloxy ligand [87,92]. In ethylene/9D1O copolymerization, the incorporation level of the polar monomer was as high as 13.6 mol% [87].

#### 2.3.1. Complexes of Group 4 Transition Metals

**Figure 8:**
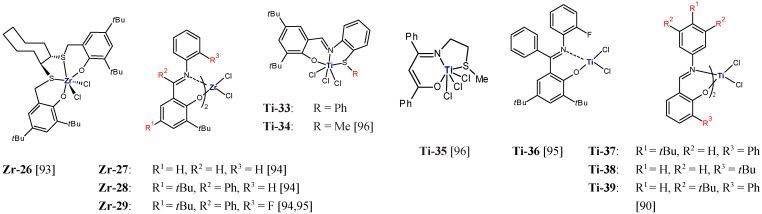
Titanium and zirconium complexes investigated as precatalysts for ethylene/α,ω-alkenols copolymerization [90,93,94,95,96].

Copolymerization of ethylene with α,ω-alkenols and their derivatives involved the use of post-metallocene catalysts based on titanium and zirconium complexes with multidentate ligands of the type [ON], [ONS], [ONNO], and [OSSO] [90,93,94,95,96,97]. Y. Saito et al. verified the ability of the zirconium complex with the [OSSO]-type bis(phenolate) ligand (**Zr-26**, Figure 8), activated by dMAO, to copolymerize ethylene with 5-hexen-1-oxytriisopropylsilane (*i*Pr_3_Si-protected 5-hexen-1-ol, 5H1OSi) [93]. That catalytic system was found to be efficient in copolymerization at 25 °C under atmospheric pressure of ethylene and it produced highly random ethylene/5H1OSi copolymers containing from 7.5 to 21.4 mol% 5H1OSi, depending on the comonomer concentrations in the feed. The highest comonomer incorporation was achieved under the most favorable process conditions, i.e., a high amount of comonomer (3.9 mmol) was used and the reaction was conducted over 5 h. The resulting copolymers had M_w_ in the range of 3800–10,800 g/mol and their dispersity was narrow (M_w_/M_n_=1.8–2.7). Unfortunately, the catalytic activity of the system decreased drastically compared with its activity in ethylene/1-hexene copolymerization, from 1110 g/(mmol_Zr_·h) to 77–470 g/(mmol_Zr_·h). That was probably due to the bulkiness of the protecting group in the monomer [93].

Good catalytic activity and incorporation ability in copolymerization of ethylene with 10-undecen-1-ol and with 9-decen-1-ol showed the zirconium catalysts **Zr-6**/Al(*i*Bu)_3_/Ph_3_CB(C_6_F_5_)_4_ [97]. The copolymerizations were carried out at various conditions and the polar monomers were subjected to pretreatment with various alkylaluminium compounds but Al(*i*Bu)_3_ was found to be the best protecting agent. Ethylene/9-decen-1-ol with the comonomer content as high as 16.4 mol% was obtained at a low comonomer concentration, 0.073 mol/L, under 1 bar of ethylene. Moreover, good comonomer incorporation (up to 5.9 mol% at the same alcohol concentration) was possible at a higher ethylene pressure (3 bar). It is also worth noting that it exhibited much higher activity in copolymerization than in ethylene homopolymerization or ethylene/1-olefin copolymerization which is contrary to most tested catalysts. 

Yang et al. employed titanium complexes **Ti-33**, **Ti-34** and **Ti-35** bearing tridentate phenoxy-imine ligands with the additional sulfur donor atom in copolymerization of ethylene and 9-decen-1-ol [96]. Copolymerizations catalyzed by **Ti-33–Ti-35**/MMAO were conducted in the presence of 20 mmol of comonomer pretreated with 1.2 equivalent of Al(*i*Bu)_3_ under 1 atm ethylene at 25 °C and at Al/Ti molar ratio of 1000. The catalytic activity of **Ti-33** and **Ti-34** was good, equal to 600 kg/(mol_Ti_·h) and 700 kg/(mol_Ti_·h), and the produced copolymers contained 3.5 mol% and 3.4 mol% of the polar comonomer. The less-hindered **Ti-35**, with the strong electron-donating ligand which improved the tolerance of active sites to functional groups, turned out more beneficial for copolymerization. It produced the copolymer with the highest activity, 10,100 kg/(mol_Ti_·h), and with the highest alcohol content, 11.2 mol%. Moreover, **Ti-35**/MMAO (Al/Ti = 3000) was able to produce the ethylene/9-decen-1-ol copolymer with high activity (1200 kg/[mol_Ti_·h]) and good comonomer incorporation (3.7 mol%), even when the comonomer was not subjected to pretreatment with Al(*i*Bu)_3_ [96]. 

The catalytic performance of the phenoxy-based zirconium complexes, **Zr-27**–**Zr-29**, activated by MAO, in copolymerization of ethylene and 10-undecen-1-ol was compared by X. Zhang et al. [94]. The reactions were carried out at 25 °C for 5 min under 1 atm ethylene. Comonomer was pretreated with Al(*i*Bu)_3_ and added in the amount of 2–16 mmol. It was found that the addition of the polar monomer caused only a very slight deactivation of the catalysts and complex **Zr-29** was the most active one. At the α,ω-alcohol concentration equal to 0.08 mol/L, its catalytic activity was equal to 19.2·10^3^ kg/(mol_Zr_·h). The results also showed that the structure of the complex was important for the incorporation of the polar monomer and molecular weights of the copolymers. Both changed in the following order: **Zr-27** < **Zr-28** < **Zr-29**. The complex **Zr-29** gave the copolymers with the highest molecular weights and the highest incorporation of polar comonomer, 3.93 wt% at 8 mmol, which increased to 8.15 wt% (1.4 mol%) at 16 mmol of comonomer in the feed. It should, however, be mentioned that the obtained incorporation level was rather low when the reaction conditions are taken into consideration. The observed changes indicate that the introduction of the phenyl group onto the imine carbon atom and introduction of the fluorine atom at the ortho position of the aniline moiety enhanced the stability of the catalyst [94]. The research on the effect of the properties of the central metal on the catalytic performance of bis(phenoxyketimine)-based complexes (**Zr-29** and **Ti-36**) in ethylene/10-undecen-1-ol copolymerization showed that **Ti-36** was less active than **Zr-29** and its ability to incorporate the comonomer was low, like that for the zirconium complex. Under the same polymerization conditions (1 atm ethylene, 12 mmol α,ω-alkenol, 10 min, 25 °C) they produced copolymers containing 6.2 wt% and 5.6 wt% comonomer, respectively [95]. When the comonomer with a shorter spacer between the polar group and the double bond was employed (5-hexen-1-ol), the catalytic activity and the comonomer content in the copolymer decreased [95].

Mono- and dinuclear zirconium complexes **Zr-14–Zr-17** were tested in ethylene/10-undecen-1-ol and ethylene/5-hexen-1-ol copolymerization over comonomer concentrations ranging from 0.02 to 0.16 mol/L in the presence of MAO as activator and Al(*i*Bu)_3_ as masking agent [98]. Modest polar comonomer incorporations, from 0.04 mol% to 1.7 mol%, were observed. 

Fujita et al. used bis(phenoxy-imine)Ti complexes, inter alia **Ti-37**, **Ti-38**, and **Ti-39**, activated by dMAO in ethylene/5- hexene-1-yl-acetate copolymerization [90]. When 1 mmol comonomer and 1 atm ethylene were applied, these catalysts produced copolymers with low comonomer incorporation, 0.13–0.90 mol%, and high molecular weights, 497,000–273,000 g/mol. It was found that the presence of the phenyl group ortho to the phenoxy-O induces higher comonomer incorporation than the *tert-*Bu group. When a higher amount of 5-hexene-1-yl-acetate was used, it resulted in the higher comonomer contents, up to 3.30 mol% for **Ti-39**/dMAO at 5.25 mmol of the comonomer in the feed.

#### 2.3.2. Complexes of Vanadium

**Figure 9:**
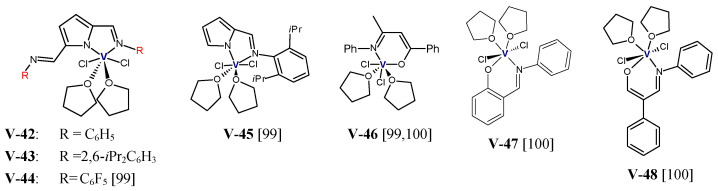
Vanadium complexes investigated as precatalysts for ethylene/α,ω-alkenols copolymerization [99,100].

L.P. Lu et al. reported the use of vanadium(III) complexes with bis(imino)pyrrolyl **V-42**–**V-44**, iminopyrrolyl **V-45**, and β-diketiminate **V-46** ligands (Figure 9) in copolymerization of ethylene with 10-undecen-1-ol [99]. The polar comonomer was subjected to pretreatment with Et_2_AlCl as a protecting reagent. The same organoaluminium compound was used as an activator, an ETA was employed as a reactivator. The reactions were conducted at 50 °C for 5 min under 1 atm of ethylene pressure and at the comonomer concentration of 0.1 or 0.5 mol/L. **V-43** was found to offer higher catalytic activity than **V-42**, and the highest activity showed **V-44**. That relation suggests that the large steric hindrance of 2,6-*i*Pr_2_C_6_H_3_ groups and interaction of fluorine atoms with hydroxyls of comonomer more efficiently protect the metal center from coordination of heteroatom and deactivation of catalyst. On the other hand, the ability of all complexes to incorporate the polar comonomer was high (12.1–14.0 mol%) and it was growing as follows: **V-43** < **V-42** < **V-44**, which demonstrates that a bulky 2,6-*i*Pr_2_C_6_H_3_ groups may make it harder for a comonomer molecule to access the active site [99]. The complexes **V-45** and **V-46** were characterized by lower tolerance towards the heteroatoms of comonomer than the bis(imino)pyrrolyl vanadium(III) complexes, but their ability to incorporate the comonomer was equally high as that for **V-44** [99]. Complex **V-46** (Figure 9) was also tested in copolymerization of ethylene with 3-buten-1-ol, 5-hexen-1-ol, and 10-undecen-1-ol. As predicted, decreasing the length of the methylene spacers in the polar comonomer decreased both the catalytic activity and comonomer incorporation [100]. In turn, the comparison of the catalytic properties of vanadium(III) complexes bearing different [N,O] bidentate ligands (**V-46**–**V-48**) revealed that their capabilities to copolymerize ethylene with 10-undecen-1-ol was only slightly dependent on the ligand structure. The catalytic activities of **V-46**–**V-48** under the same reaction conditions were 9.72, 8.70, and 11.2 kg/(mmol_v_·h), while the 10-undecen-1-ol incorporation levels in the resultant copolymers were 3.70, 4.00, and 3.40 mol%, respectively [100]. Selected results of ethylene/α,ω-alkenols copolymerization with vanadium and group 4 metal complexes were displayed in Table 7.

## 3. Conclusions

The catalytic copolymerization of ethylene with other vinyl monomers like 1-olefins, styrenic and polar monomers, produces materials with unique and interesting set of properties which can fill property gaps not addressed by HDPE. Numerous efficient post-metallocene catalysts based on well-defined complexes of both vanadium and group 4 metals have been developed for ethylene/1-olefin copolymerization. The wide variation of the available structures of the complexes allows the production of copolymers which differ with regard to their composition, chemical composition distribution, molecular weight, and its distribution. It is worth noting that vanadium post-metallocene catalysts often afforded more homogeneous copolymers than their group 4 metal counterparts. Moreover, development of the catalysts, which exhibit living behavior and chain shuttling systems, allowed for the formation of block and multi-block copolymers with various compositions and microstructures. From the work described herein, it is also clear that the successful copolymerization of ethylene with styrene and/or its derivatives was achieved only in the presence of a few titanium and zirconium complexes, while any post-metallocene vanadium complexes were not applicable in that process. As for the copolymerization of ethylene with α,ω-alkenols, complexes of both group 4 and group 5 metals were tested. However, their catalytic activity was usually much lower compared to that for homopolymerization of ethylene, despite the protection of hydroxyl group. Moreover, the reaction conditions for the reported copolymerizations were selected to provide the highest possible incorporation of comonomer. Namely, the process was conducted at atmospheric pressure or lower, which is favorable indeed for higher comonomer contents, but which considerably reduces catalytic activity and molecular weight of the copolymer product. The one exception was the catalytic system based on diamine-bis(phenolate) zirconium complex (**Zr-6**) for which a significant activating effect of the polar comonomer on the catalyst activity was observed.

## Figures and Tables

**Table 1:** Classification of linear polyethylene by its weight [11].

PE Type	M_w_, kg/mol	T_m_, °C
HDPE	100–500	125–135
HMWPE	200–1000	130–135
UHMWPE ^2^	>3000 ^1^	135–148

HMWPE—high molecular weight polyethylene, UHMWPE—ultra high molecular weight polyethylene, ^1^—viscosity average molecular weight (M_v_), and ^2^—there is no single definition for that polymer. According to ISO 11542-1, this is PE with molecular weight of at least one million g/mol, while ASTM D 4020-00a specifies that the molecular weight value for UHMWPE is over 3.1 million g/mol [15].

**Table 2:** Structures of main grades of polyethylene and ethylene copolymers.

Ethylene Homopolymers		Ethylene Copolymers		
**HDPE**	**UHMWPE**	**LLDPE**	**mLLDPE**	**VLDPE**
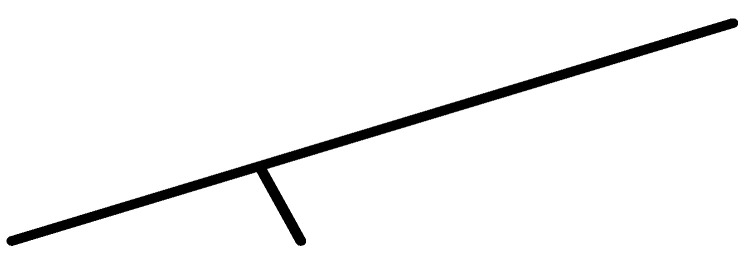	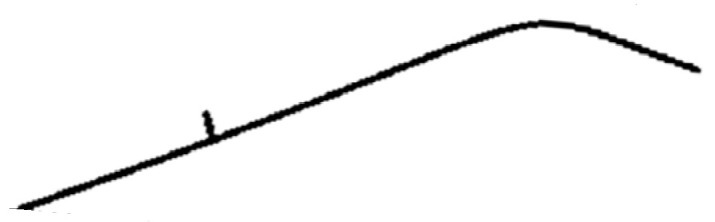	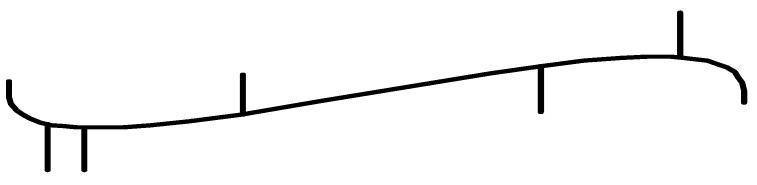	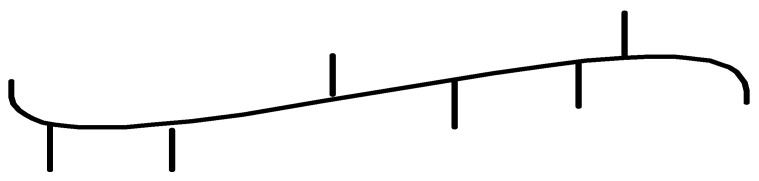	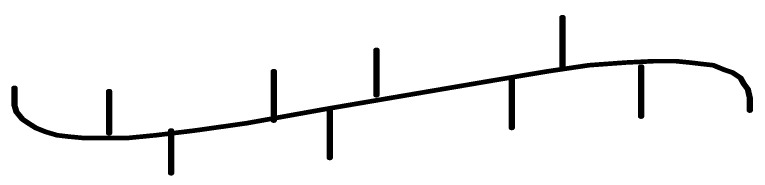
**LDPE**		**ULDPE**	**OBC**	**COC**
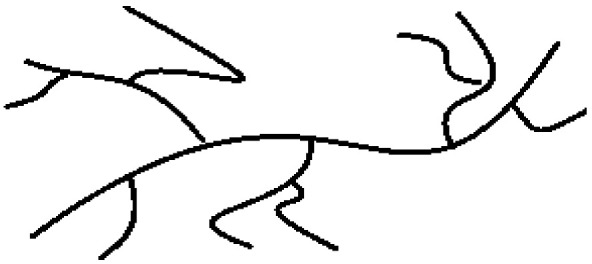		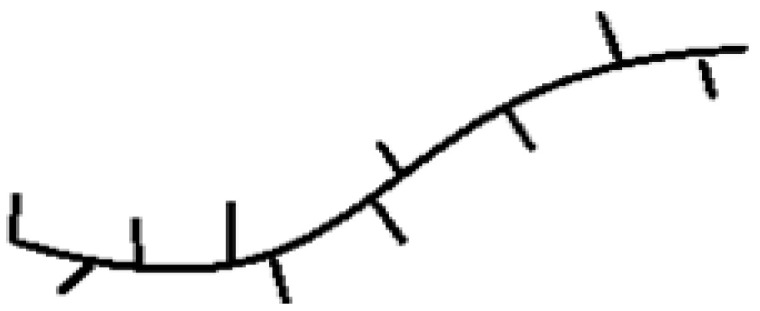	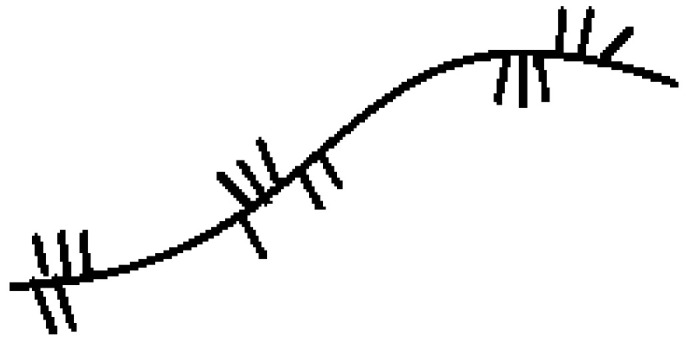	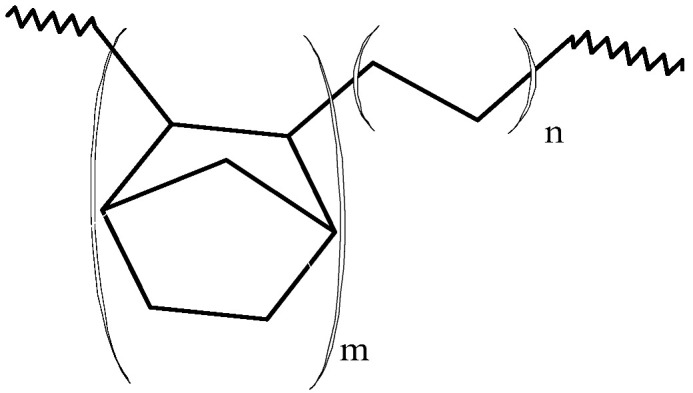

**Table 3:** Properties of LDPE and main polyethylene and ethylene copolymers grades produced using transition metal catalysts.

Type of (Co)Polymer	*d*^1^, g/cm^3^	*T_m_*^2^, °C	*χ*^3^, %	MW ^4^, kg/mol	CC ^5^, wt%	Ref.
**UHMWPE**	0.93–0.94	130–140	60–75	2000–6000	-	[23]
**HDPE**	0.94–0.97	130–135	60–80	200–500	0–2.5	[23,24]
**LDPE**	0.91–0.93	100–110	40–50	≤600	0	[23,24]
**LLDPE**	0.91–0.94	120–125	40–60	≤600	5.0–12.0	[23,24,25]
**VLDPE**	0.89–0.91	118–122	25–40	-	10.0–35.0	[24,25]
**ULDPE**	0.86–0.89	40–84	-	-	-	[26,27]
**OBC**	~0.865–0.935	~120–130	-	-	-	[25]
**COC**	1.01–1.02	100–180 ^6^	-	-	-	[23]

^1^*d*—density, ^2^*T_m_*—melting temperature, ^3^*χ*—crystallinity, ^4^ molecular weight, ^5^ CC—comonomer content in copolymer, and ^6^ glass temperature (*T_g_*, °C).

**Table 4:** Results of ethylene/1-olefin copolymerization with group 4 metal complexes.

Complex	[M], µmol	Activator	E, bar	Time, min	T, °C	Comonomer		Activity, kg/(mol_M_·h)	CI, mol%	M_w_, kg/mol	M_w_/M_n_	Ref.
						Type	mol/L					
Zr-2	5	MMAO	2.8	^1^	20	1-Hex	0.0666 ^2^	798	49.9	209 ^3^	1.7	[29]
Zr-4	100	MMAO	3.6	^1^	20	1-Hex	0.0809 ^2^	460	64.1	114 ^3^	1.6	[29]
Zr-6	80	Al(*i*Bu)_3_/B	5	30	60	1-Oct	0.19	405.0	6.5	42	2.1	[30]
Zr-6 ^4^	50	Al(*i*Bu)_3_/B	5	30	60	1-Oct	0.27	28.3	39.5	15.8	5.5	[31]
Ti-1	80	Al(*i*Bu)_3_/B	5	30	60	1-Oct	0.19	63.8	2.7	235	2.4	[30]
Ti-4	80	Al(*i*Bu)_3_/B	5	30	60	1-Oct	0.19	44.8	3.2	292	3.9	[30]
Ti-4	80	Al(*i*Bu)_3_/B	5	30	60	1-Oct	0.70	42.0	12.0	755	1.7	[30]
T-5	70	MAO	5	30	30	1-Oct	0.73	78.2	3.7	229	17.6	[32]
Ti-8	7	MMAO	1	15	25	1-Hex	0.7	23.0	14.1	229	2.5	[35]
Ti-9	10	MMAO	1	15	25	1-Hex	0.7	387.0	30.0	146	2.36	[35]
Ti-10	3	MMAO	1	60	50	1-Hex	0.34	100.0	11.0	79	2.0	[36]
Ti-11	3	MMAO	1	10	50	1-Hex	0.34	220.0	0.6	143	3.8	[36]
Ti-12	25	Al(*i*Bu)_3_/B	5	20	60	1-Oct	0.58	87	3.8	690	26.5	[37]
Zr-9	10	AlMe_3_/B	4	2	40	1-Oct	0.64	2846 ^5^	7.2	6.0	1.5	[38]
Zr-10	10	AlMe_3_/B	4	0.5	40	1-Oct	0.64	5649 ^5^	1.0	5.0	1.3	[38]
Hf-1	3 ^6^	B(C_6_F_5_)_3_	1	30	25	1-Hex	0.1	5.4	25	5.6 ^3^	1.31	[41]
Hf-1	3 ^6^	B(C_6_F_5_)_3_	1	30	25	1-Hex	0.5	20.3	72	14 ^3^	1.18	[41]
Hf-6	1	Al(*i*Bu)_3_/B	10	10	80	1-Hex	1.0	5280	8.7	931	4.3	[44]

1-Hex = 1-hexene, 1-Oct = 1-octene, E = ethylene, CI = comonomer incorporation, B = Ph_3_CB(C_6_F_5_)_4_, ^1^ 20–30 min, ^2^ feed ratio of comonomers X_E_/X_1-Hex_, ^3^ M_n_, kg/mol, ^4^ copolymerization in the presence of AlMe_3_ (AlMe_3_/Zr = 100), ^5^ kg/(mol_M_·h·atm), and ^6^ mmol/L.

**Table 5:** Results of ethylene/1-olefin copolymerization with vanadium complexes.

Complex	[V], µmol	Activator	ETA/V	E, bar	Time, min	T, °C	Comonomer		Activity, kg/(mol_V_·h)	CI, mol%	M_w_·10^3^, g/mol	M_w_/M_n_	Ref.
							Type	mol/L					
V-1	0.5	Et_2_AlCl	500	1	5	25	1-Hex	0.20	4800	4.20	25.9	1.86	[57]
V-3	0.5	Et_2_AlCl	500	1	5	25	1-Hex	0.60	5040	8.80	20.7	1.76	[57]
V-3	0.5	Et_2_AlCl	500	1	5	75	1-Hex	0.60	3380	16.1	3.2	1.80	[57]
V-14	1.0	Et_2_AlCl	300	1	10	25	1-Hex	0.20	1200	3.44	38.5	2.4	[59]
V-16	1.0	Et_2_AlCl	300	1	10	25	1-Hex	0.20	2580	2.92	52.1	1.8	[59]
V-18	1.0	Et_2_AlCl	300	1	10	50	1-Hex	0.20	5100	3.45	28.2	1.7	[59]
V-25	1.0	Et_2_AlCl	300	1	10	25	1-Hex	0.27	3180	3.00	56.4	2.0	[60]
V-25	1.0	Et_2_AlCl	300	1	10	25	1-Hex	1.35	900	12.9	24.5	1.9	[60]
V-33	8.0	EtAlCl_2_	-	5	30	30	1-Oct	0.19	350	4.1	99.0	2.3	[62]
V-33	8.0	EtAlCl_2_	-	5	30	30	1-Oct	0.38	155	6.7	-	-	[62]
V-37	10.0	EtAlCl_2_	-	2	30	ng^1^	1-Hex	1/800 ^2^	120	7.4	71.0	1.7	[64]
V-39	10.0	EtAlCl_2_	-	2	30	ng^1^	1-Hex	1/800 ^2^	167	7.5	2882	225.5	[64]

1-Hex = 1-hexene, 1-Oct = 1-octene, E = ethylene, CI = comonomer incorporation, ^1^ ng—temperature was not given, and ^2^ catalyst/1-hexene = 1/800.

**Table 6:** Ethylene/styrene copolymerization results.

Complex	[M], µmol	Activator	E, bar	Styrene, mol/L	Activity, kg/(mol_Mt_·h)	*T_m_*, °C	χ, %	CI, mol%	Ref.
Ti-21	4	MAO	2.56	1.1	52 ^1^	-	-	1.2	[79]
Ti-22	20	MAO	2.56	1.1	10 ^1^	-	-	10.0	[79]
Ti-23	20	MAO	2.56	1.1	1 ^1^	-	-	35.2	[79]
Ti-26	0.1 ^2^	MAO	0.20 ^3^	2.2	1600	30.5 ^4^	-	55.0	[80]
Ti-26	0.08 ^2^	MAO	0.20 ^3^	0.55	1300	40.5 ^4^	-	40.0	[80]
Ti-1	10	MMAO	5	0.55	28.8	125.0	41.3	0.5	[86]
Zr-6	10	MMAO	5	0.8	475.6	110.8	57.7	3.0	[86]

E = ethylene, CI = comonomer incorporation, ^1^ activity in kg/(mol_M_·h·mol/L), ^2^ mmol/L, ^3^ mol/L, ^4^ *T_g_*.

**Table 7:** Ethylene/α,ω-alkenols copolymerization results.

Complex	[M], µmol	Activator	E, bar	Time, min	Comonomer		Activity, kg/(mol_M_·h)	CI, mol%	M_w_, kg/mol	M_w_/M_n_	Ref.
					Type	mmol					
Zr-26	2	dMAO	1	60	5H1OSi	2.3	121	7.5	3.8	2.7	[93]
Zr-26	2	dMAO	1	300	5H1OSi	3.9	77	21.4	10.2	1.9	[93]
Zr-6	10.0	Al(*i*Bu)_3_/B	1	20	9-D-1-ol	6.75	45.3	16.4	2.9	1.6	[97]
Zr-6	10.0	Al(*i*Bu)_3_/B	3	20	9-D-1-ol	6.75	604.2	2.9	4.7	1.9	[97]
Ti-33	3.5	MMAO	1	10	9-D-1-ol	20.0	600	3.5	940	2.9	[96]
Ti-34	3.5	MMAO	1	10	9-D-1-ol	20.0	700	3.4	115	2.2	[96]
Ti-35	3.5	MMAO	1	1	9-D-1-ol	20.0	10100	11.2	178	2.2	[96]
Zr-29	1.0	MAO	1	5	10-U-1-ol	16.0	13100	1.4	-	-	[94]
V-42	0.5	Et_2_AlCl	1	5	10-U-1-ol	5.0	8160	4.2	14.3	1.8	[99]
V-42	0.5	Et_2_AlCl	1	5	10-U-1-ol	25.0	2160	13.7	3.8	1.9	[99]

E = ethylene, CI = comonomer incorporation, B = Ph_3_CB(C_6_F_5_)_4._
